# Social Media Role and Its Impact on Public Health: A Narrative Review

**DOI:** 10.7759/cureus.33737

**Published:** 2023-01-13

**Authors:** Sushim Kanchan, Abhay Gaidhane

**Affiliations:** 1 Epidemiology and Public Health, Jawaharlal Nehru Medical College, Datta Meghe Institute of Medical Sciences, Wardha, IND

**Keywords:** hipaa violation, web 2.0, social media platforms, public health, social media (sm)

## Abstract

Social media refers to online social networking sites and is a broad example of Web 2.0, such as Twitter, YouTube, TikTok, Facebook, Snapchat, Reddit, Instagram, WhatsApp, and blogs. It is a new and ever-changing field. Access to the internet, social media platforms and mobile communications are all tools that can be leveraged to make health information available and accessible. This research aimed to conduct an introductory study of the existing published literature on why to choose and how to use social media to obtain population health information and to gain knowledge about various health sectors like disease surveillance, health education, health research, health and behavioral modification, influence policy, enhance professional development and doctor-patient relation development. We searched for publications using databases like PubMed, NCBI, and Google Scholar, and combined 2022 social media usage statistics from PWC, Infographics Archive, and Statista online websites. The American Medical Association (AMA) policy about Professionalism in Social Media Use, American College of Physicians-Federations of State Medical Boards (ACP-FSMB) guidelines for Online Medical Professionalism, and Health Insurance Portability and Accountability Act (HIPAA) social media violations were also briefly reviewed. Our findings reflect the benefits and drawbacks of using web platforms and how they impact public health ethically, professionally, and socially. During our research, we discovered that social media's impact on public health concerns is both positive and negative, and we attempted to explain how social networks are assisting people in achieving health, which is still a source of much debate.

## Introduction and background

The term "social media" was first used to describe the evolution of Web 2.0 applications that are open and social in nature [1]. Web 2.0 social networking sites are broad online platforms where people can communicate and share information and as we enter the digital age, this media platform is becoming more popular. With 3.81 billion active social media users in April 2020 [1,2], increasing access to the internet and mobile phone connections, more people have access to public health information more quickly and directly than ever before.

**Figure 1:**
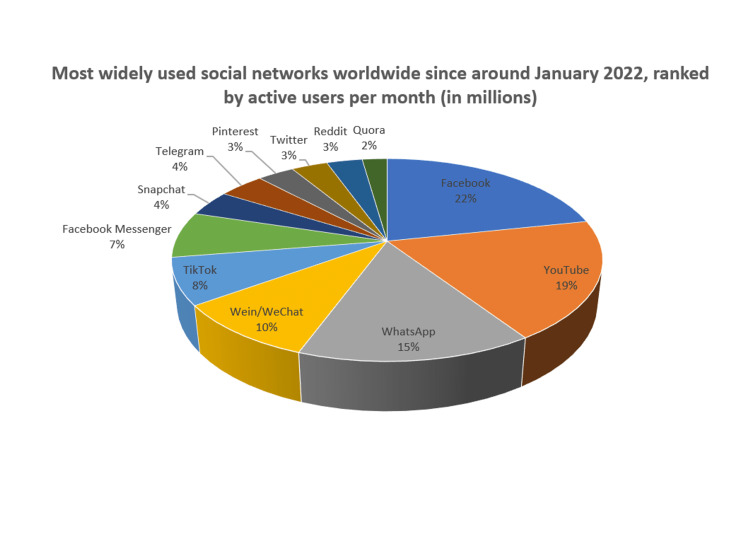
Most widely used social networks worldwide since around January 2022, ranked by active users per month (in millions); in percentage. [4]

In the world of social media, 2020 was extremely important, world's most popular social media website, Facebook, has 1.1 billion monthly users [3] in 2013 which got increased to 2.9 million by 2022 [4]. Globally, there are more than 3.6 billion users of social media, and by 2025, that number is projected to increase to 4.41 billion [5]. It was found that YouTube is the second most actively used networking site after Facebook, with 2,562 million users in 2022. WhatsApp had 2000 million, Wein/WeChat - 1,263; TikTok - 1,000; Facebook Messenger - 988; Snapchat - 557; Telegram - 550; Pinterest - 444; Twitter - 436; Reddit - 430, and Quora - 300 million active users. Figure 1 is a percentage-based compilation of global usage data from January 2022 for all mentioned social media networks.

For several reasons, online social media platforms appear to have much potential for public health campaigns. For instance, they can connect with very reasonably large audiences, Facebook has 1.1 billion monthly users [3] in 2013. Second, messages can be sent to personal contacts, possibly making them more advantageous than traditional health marketing tactics [6]. Third, user involvement and retention are typically high on online social networks in contrast to conventional web-based interventions [7]. Finally, because social media involves users taking an active role and creating content, it can be more impactful than traditional websites [8]. Various studies provided an overview of social media's potential as a tool for health interventions, socializing with supportive friends and family, talking about your emotions, healthy behavior change and counseling, health campaigns, medical education, disease outbreak surveillance, health research, and more [9]. These recent developments contemplate how social media offers healthcare professionals and patients opportunities to communicate affordably and reciprocally, which can positively impact current medical practice.

Despite the promising strategies Web 2.0 technologies and eHealth applications provide, it raises many questions, establishing trust, adhering to rules, and choosing the best content are just a few of these [10]. A lot of user-generated content (UGC) from self-media and various facts about the epidemic on social media have a significant emotional undertone [11]. It shed light on the patterns and characteristics of how users' emotional dispositions change during times of public health emergency [11], and how social networking can influence people's and groups' decision-making behavior [12], potentially increasing the risk of misinformation, various conspiracy theories, stigma, violence, and religious-cultural sentiments damage. Overusing social media has been linked to significant issues with the mental health of both adults and adolescents. Fear of missing out (FOMO) is the anxiety associated with the motivation to keep up with what other people are doing on social media. Cyberbullying, sleep disruption, stress, depression, and false prestige are just a few of the negative effects of social media on today's youth. Other challenges that can arise are offending people and defaming their relationships and reputations, either unintentionally or intentionally [13]. It can be difficult to use numerous social networking sites for medical purposes to improve communication because one must be sure that the information is accurate and easily accessible [10]. Due to concerns with compliance, trust, and patient privacy, social media has been warned about having a significant negative impact on doctor-patient relationships [14]. However, the accepted protocols for using web networks to transmit health information have not yet been investigated. Another topic that has to be investigated is how people view and use personal health data and cultural and social standards that vary by region.

This narrative study intends to shed light on the potential use of social media as a new platform for the population health and healthcare industries. It was also emphasized that it was important to examine the many difficulties that could arise when using this platform for the health sectors and to provide guidelines on certain key social media usage best practices.

## Review

**Figure 2:**
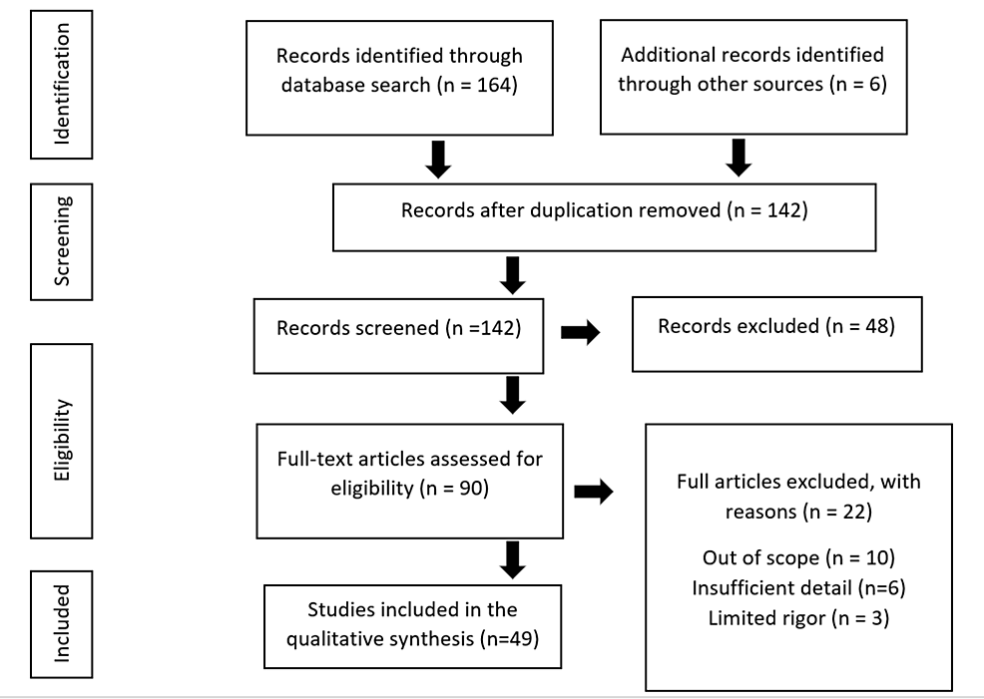
Inclusion and exclusion criteria of this study. The image is created by the author (ASR) of this study.

**Figure 3:**
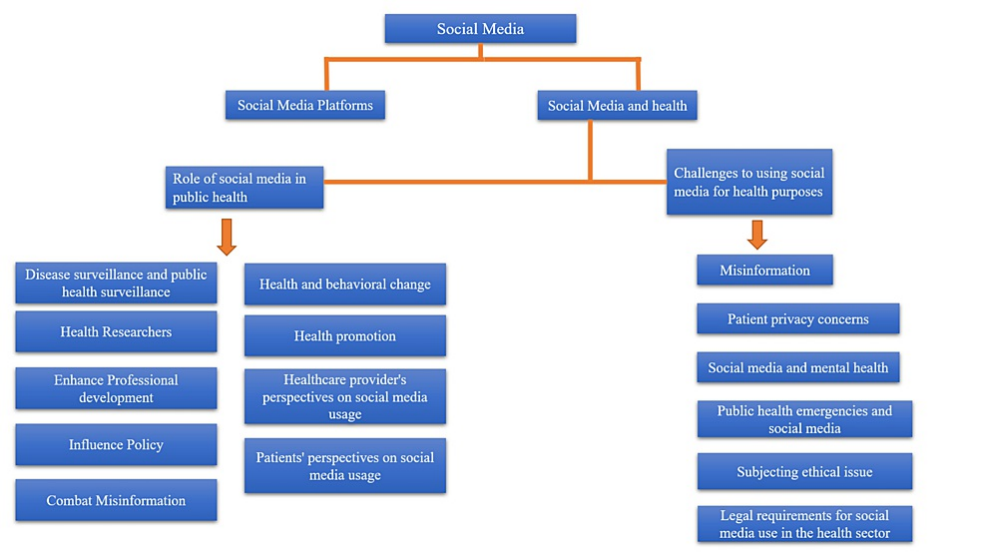
Summary status of this study. The image is created by the author (ASR) of this study.

The topic "Social Media Role and Its Impact on Public Health" was thoroughly researched using databases and websites for up-to-date related data and literature, such as PubMed, NCBI, and Google Scholar. Search terms included social media, social networking, public health, online health information, online health communication, online health management, social media platforms, social media usage statistics, HIPAA violation, and legal and ethical standards. In addition, an online search was conducted using a search engine such as Google to discover health sites data from five portals and websites including 2022 social media usage statistics from PWC, Infographics Archive, and Statista online domains on some of the most well-known social media toolkits. Figure 2 shows the inclusion and exclusion criteria of this study and Figure 3 shows the summary status of this study.

Role of social media in public health

Disease Surveillance and Public Health Surveillance

Social-networking sites for clinicians, patients, and the general public hold potential for harnessing the collective wisdom of the masses for public health surveillance. Organizations like the Global Outbreak Alert and Response Network of the World Health Organization (WHO) also relies on web sources for up-to-date surveillance activities as data were not captured via any traditional method [15]. A 2018 study conducted by Yasmin and her team examined geolocated tweets for public health surveillance during a mass gathering in Canada and compare Twitter data against other data sources for heat alerts during the 2015 Pan/Parapan American Games. His study stated that Syndromic surveillance uses pre-diagnostic data, and the inclusion of syndromic data sources in public health surveillance for mass gatherings has been shown is useful [16].

The platform has an opportune disease surveillance area, improving its capability to detect disease outbreaks. Media and techniques known as user-generated information and real-time information surveillance of various public health outcomes, such as influenza, foodborne illnesses, or heat alerts, can identify cases of infectious diseases more quickly, which in the case of alerts, may permit investigation or action. A study conducted by Wakamiya et al. in 2018 using Twitter to detect Influenza outbreaks via geotagging tweets and trapped sensors supported the evidence [17]. Another study, Platform for Automated Extraction of Disease Information from the Web, by Arsevska et al., developed a platform to detect automatically animal infection outbreaks in France from their online news sources (PADI-web). Data were retrieved from 4,500 news websites, including Google News [18]. This paper serves as an excellent illustration of how such a web-based system has been fully implemented and evaluated. Using text categorization, authors Effland et al. created a system for finding foodborne illnesses reported in Yelp restaurant reviews. The New York City Department of Health and Mental Hygiene (DOHMH) uses the system to track concerns about foodborne illnesses on Yelp [19].

Health Researchers

Studies have shown that social media is used by health researchers for a variety of research-related goals. The platform is most frequently utilized to find participants and get data from the Internet (e.g., content analysis of social media posts and data mining on social media) [20]. It also helps in networking with colleagues and knowledge users, to distribute public health research, for example, sharing links about scientific publications or research on social media can help to broaden readership, exponentially increasing reach [21]. We identified the three most popular social media platforms in 2020; Facebook, Twitter, and Instagram, as the major social media platforms in use for health research [22]. Professional associations, public health organizations (such as WHO and the Centers for Disease Control and Prevention (CDC)), and hospitals frequently communicate via social media about science and health [21]. Social media is used by all major news organizations, giving additional distribution channels and ways to mix current events with smartphone capabilities [21].

Enhance Professional Development

There are many opportunities for professional engagement outside of conventional contexts due to the growing social media presence of academics, physicians, business professionals, public health departments, and healthcare systems. Public health experts can interact with the public through several Twitter chats, including CDC chats [21].

Influence Policy

Sharing arguments in favor of or against health policies with the general public, decision-makers, and other important stakeholders is made possible by social media. Using social media to inform constituents about proposed laws and encouraging them to contact political representatives to voice their ideas can have an impact on politicians' behavior because politicians are driven to please their voters. Social media is becoming more and more important in discussions of politics and policy, as evidenced by the president's massive Twitter following and usage of the platform to interact directly with the people [21].

Combat Misinformation

Suppose public health professionals are more active on social media. In that case, it may be possible to mitigate the impacts of people making false claims and to boost fact-checking initiatives by making more accurate online health information available. Hence, social media interaction with experts might help dispel incorrect information [21].

Health and Behavioral Change

In a study released in 2022 by Bonar et al., the authors employed social media adverts to target young people who engaged in risky drinking and came up with some encouraging findings [23]. The prevalence of cannabis use among emerging adults (aged 18 to 25) necessitates preventive measures. Bonar et al. conducted another study in 2022 to develop an eight-week persuasive questioning and behavioral intervention focusing on cannabis usage among emerging adults using the unique platform of social media [24].

Health Promotion

The included research showed that various social media outlets could raise the degree of women's health promotion [25], awareness of menstrual hygiene, understanding of breast cancer awareness [26], breastfeeding techniques [27], and adherence, self-perception and promotion of oral health [28,29], significant use of antibiotics [30], consistency with exercise, sexual health promotion [31], road safety awareness [32], smoking cessation, adverse drug reaction reporting [33], and many more in a row.

Healthcare Provider’s Perspectives on Social Media Usage

Healthcare professionals (HCPs) are constantly looking for better and more effective ways to reach greater audiences, particularly those who were difficult to reach through conventional techniques. This social networking platform offers professionals for health promotion with cost-effective opportunities to advance their careers by building communities of professionals, participating in professional development activities, and meliorating classroom learning [34]. These interactive tools and platforms are already commonplace in clinical settings, and many practitioners use them to connect with their target audience on both a personal and professional level [34].

Patients’ Perspectives on Social Media Usage

In social media, there are 74% of Internet users, and 80% of those use social media to research doctors, hospitals, and medical news and information [35]. Consumers on social media who view health-related consumer reviews are 42% and 32% of users share their friends' or family members' health experiences (PWC) [36]. In the most popular use social platform Facebook, 28% of health conversations support a health-related cause (from the Infographics Archive) [36]. Susannah Fox, Chief Technology Officer at the U.S. Department of Health and Human Services, refers to this emerging trend as "peer-to-peer health care," explaining that "patients are willing to share what they know, related to health, treatments, sources, facilities." "Peer-to-peer health care" is described by Fox as "the most exciting innovation in health care today" [13].

Due to patients' improved knowledge of health information and their increased involvement in maintaining their health, social media has indisputably altered the relationship between patients and practitioners. Other areas where social media can be helpful for patients include identifying health professionals, peer support and sharing experiences, promoting healthy behavior, and so on [37]. It may improve health outcomes by facilitating communication about health issues between general health professionals, patients, and the public.

Table 1 enlists studies conducted all over the world on the use of social media as a tool in various health sectors.

**Table 1:** List of studies conducted all over the world on the use of social media as a tool in various health sectors.

S. No.	Authors	Year	Studies/findings	Health sectors
1	Bonar et al. [23]	2022	Interventions using social media to prevent risky drinking in young adults and adolescents	Health behavioral change
2	Bonar et al. [24]	2022	A social media campaign to reduce cannabis consumption among young adults	Health behavioral change
3	Leong et al. [38]	2022	Improve Type 2 Diabetes Patients' Self-Management and Attitudes During the COVID-19 Pandemic via social media-Delivered Patient Education	Medical Education
4	Mattingly, T. Joseph [39]	2015	Using social media for creative patient care	Medical Education
5	AlSadrah, Sana A. [40]	2021	Use of social media in the Gulf Cooperation Council to promote public health	Health promotion and education
6	Stellefson et al. [41]	2020	Social Media's Changing Role in Health Promotion	Health promotion and education
7	Kesten et al. [31]	2019	distributing information on social media to promote sexual health	Health promotion and education
8	Bulcock et al. [33]	2021	Improve Adverse Drug Reaction Reporting	Health promotion and education
9	Veerappan et al. [32]	2022	Road traffic safety awareness	Health promotion and education
10	Sharma et al. [28]	2022	Oral Health Hygiene	Health promotion and education
11	Dewi et al. [26]	2022	Breast self-examination practice	Health promotion and education
12	Munyan et al. [27]	2022	Promoting breastfeeding	Health promotion and education
13	Zucco et al. [30]	2018	Using social media to look for information about antibiotics	Health promotion and education
14	Breland et al. [21]	2017	Utilizing Social Media to Broaden the Impact of Public Health Research	Health research
15	Dol et al. [20]	2019	Social media use by Health Researchers	Health research
16	Aiello et al. [42]	2020	Internet and social media for Disease Surveillance in Public Health	Disease surveillance

Challenges to using social media for health purposes

Misinformation

The longest impediment to the internet, in general, is that it's open to everyone; anyone can post information on any topic they want [10]. This turns people into self-appointed experts and (knowingly or unknowingly) spreads false information, certain online information can have different points of view and vary depending on geographical and cultural factors [10]. These conflict-causing situations are tactics to deal with, and how users sharing that information can be protected are some of the digital era’s other challenges.

Patient Privacy Concerns

Due to data confidentiality concerns, some patients are hesitant to share information via the web platform [36]. Posting distinguishable health information on these platforms without concern for patients would undoubtedly create unfaith and overstep their privacy boundary, as well as question occupation as a whole. If the staff does not follow the necessary guidelines, social media can lead to HIPAA violations (Health Insurance Portability and Accountability Act) [36]. In some cases, the content posted by healthcare professionals on their social accounts was so bad that the provider who published the content that drew criticism left the platform altogether - Reputational Harm [10].

Social Media and Mental Health

Although the majority of college students use social media without incident, a minority percentage of users engage in excessive or compulsive behavior on these platforms. Problematic social media use is a behavioral addiction defined by excessive worry over online activities, uncontrollable cravings to access or use social media, and spending so much time and energy on social media that it has a negative influence on significant parts of one's life. In India, 19.9% of college students that use social media have problematic social media [43]. It is understandable that parents, policymakers, and researchers all want to know how adolescents' frequent use of social media affects their mental health because it gives them numerous opportunities to engage in risky behaviors, join questionable communities, and interact with strangers without parental supervision [44].

Public Health Emergencies and Social Media

People and communities experience stress during public health emergencies. It is challenging to disseminate official public health information on infectious diseases because people frequently get information from social contacts through personal interactions or social media, subject to bias and misunderstanding. Misinformation during the Ebola outbreak in the Democratic Republic of the Congo in 2019 was associated with aggression, mistrust, social unrest, and targeted assaults on healthcare workers [45]. During the SARS outbreak in China in 2002-2003, Asian people faced social stigma as a result of their fear of contracting the disease [45]. Rumors about COVID-19 have been labeled a global enemy by the UN secretary-general [45]. Although debunking research has demonstrated that well-designed corrections can reduce the effects of false information, nothing is known about the effects of correction in the context of protracted social media arguments [46].

Subjecting Ethical Issue

Patients must be empowered with accurate and up-to-date information about their health to make fully informed treatment decisions, as their autonomy should be valued [47]. Physicians should be held accountable for using the powers endowed upon them by the patient's trust as trust is a pillar of the medical profession [47], and their healthcare practice should be motivated by good intentions at all times. Indeed, when using social media to connect with patients directly or share information, physicians as healthcare professionals should always consider the proper measure while communicating or sharing content [47]. When using social media, one should be mindful of the rules and ethical considerations. Information subjecting to harm someone's beliefs, norms, or any religious concerns is another major issue.

Legal Requirements for Social Media Use in the Health Sector

Guidelines are especially useful in new and evolving areas. If information or practices turn out to be incomparable, guidelines are created to connect them [2]. The guidelines aim to suggest, discover, and guide learners through questionnaires [2]. Medical schools have taken disciplinary action against amateurish digital information posted by medical students, including dismissal in some cases [48]. In November 2010, the American Medical Association (AMA) issued a policy statement about how professionals should use social media channels cautiously, separating professional and personal profiles and keeping patient details private [48]. Similarly, the Accreditation Council for Graduate Medical Education (ACGME) outlines some similar areas [49]. The American College of Physicians-Federations of State Medical Boards (ACP-FSMB) guidelines on Online Medical Professionalism state that guidelines are a baseline and starting point which needs to be evolved or restructured timely by parallelly adopting advanced technologies and eventually emerging with best practices [2]. They also worked on social media and web networking usage guidelines [2]. Nevertheless, there are still concerns about maintaining professional boundaries when using social media. In addition, there are still no agreements on what constitutes professional Internet behavior, except for the most horrific mistakes in professional unethical and illegal activities [49]. Even though it is difficult to measure and teach professionalism objectively, progress is being made in areas such as confidential patient details, pharmaceutical companies’ involvement details, ethics, and a lawsuit, skills of interaction, and portable health insurance [49].

Mix influence

Social media platforms allow for the exchange of health-related information, health promotion, policy influence, the development of relationships between healthcare practitioners and patients, the identification of drug misuse or misunderstandings among the general public, the dissemination of accurate information, and the collection of concurrent health data. This platform is used by public health organizations such as the WHO's Global Outbreak Alert and Response Network for real-time surveillance [15]. It appears to be a useful platform for health researchers looking to recruit participants and collect data from the Internet. These online platforms appear to be a useful way of providing behavioral counseling, lending credence to the idea that social networking influences individual and group decision-making. Trust, compliance, and knowledgeable content should be prioritized for social media to have an impact on the population, which in turn has an impact on public health. More research is needed to determine how to promote healthy behaviors and collect and disseminate reliable information using these tools. False-positive data, on the other hand, continues to impede the accuracy of the Internet-based monitoring system. Patient privacy concerns, as well as religious-cultural sentiments, can all be easily violated as a result of an undefined policy of using social media to spread violence and disbelief. Social media acts as a quick platform during a public emergency for disseminating rumors, exposing false information and conspiracy theories, and escalating fear and stigma directed at specific people and locations. Numerous challenges, including authority, professionalism, confidentiality, customs, information quality, and secrecy, as well as the tremendous role that social networks play in medical and public health care, remain unaddressed.

## Conclusions

Our findings suggest that social media is an emerging platform with numerous opportunities for us to use it in public health and that it has an impact on the relationship between physician and patient, public trust in the system, and potential lawsuits, as well as changes in various health sectors such as health interventions, behavioral modification and promotion, health campaigns, medical education, disease outbreak surveillance, health research, and more. Because of the two ends of the spectrum, our analysis shows that, while social media can be a powerful tool for the public health sector in the current digital era, there are also drawbacks to consider. These booming platforms are not exempt from these drawbacks, which include potential moral, ethical, legal, and privacy violations, professional behavior concerns, compliance-related issues, and societal ramifications. In addition, some major ethical issues are discussed briefly, the AMA policy about professionalism in social media use, ACP-FSMB guidelines for Online Medical Professionalism, and HIPAA social media violations are used to present certain proposed regulations and guidelines for using social media for the population's health, which may be applied for avoiding such consequences.

To summarize, even if there are multiple issues, risks, and dangers, we can overcome these obstacles and utilize technology to its fullest extent if problems are addressed, acknowledged, and tried to be eliminated. Focusing on how we might use social media and its attendant demands is both necessary and ethical because it may be difficult to achieve continuous growth and evolution without setting adequate criteria and regulations for doing so. The limited and conflicting results of critical evaluations of previously published research on the influence of social media on public health issues give credibility to this argument. Our research indicates that the use of social media in public health has conflicting results, and it is advised that more research be done in this area.
